# Image-Based Dynamic Phenotyping Reveals Genetic Determinants of Filamentation-Mediated β-Lactam Tolerance

**DOI:** 10.3389/fmicb.2020.00374

**Published:** 2020-03-13

**Authors:** Taiyeb Zahir, Dorien Wilmaerts, Sabine Franke, Bram Weytjens, Rafael Camacho, Kathleen Marchal, Johan Hofkens, Maarten Fauvart, Jan Michiels

**Affiliations:** ^1^Centre of Microbial and Plant Genetics, KU Leuven, Leuven, Belgium; ^2^VIB-KU Leuven Center of Microbiology, Leuven, Belgium; ^3^Department of Information Technology, IDLab Group, Ghent University, Ghent, Belgium; ^4^Department of Plant Biotechnology and Bioinformatics, Ghent University, Ghent, Belgium; ^5^Department of Chemistry, KU Leuven – University of Leuven, Leuven, Belgium; ^6^Interuniversity Microelectronics Centre (IMEC), Leuven, Belgium

**Keywords:** β-lactam, antibiotic tolerance, filamentation, high-throughput microscopy, bacteriolysis

## Abstract

Antibiotic tolerance characterized by slow killing of bacteria in response to a drug can lead to treatment failure and promote the emergence of resistance. β-lactam antibiotics inhibit cell wall growth in bacteria and many of them cause filamentation followed by cell lysis. Hence delayed cell lysis can lead to β-lactam tolerance. Systematic discovery of genetic factors that affect β-lactam killing kinetics has not been performed before due to challenges in high-throughput, dynamic analysis of viability of filamented cells during bactericidal action. We implemented a high-throughput time-resolved microscopy approach in a gene deletion library of *Escherichia coli* to monitor the response of mutants to the β-lactam cephalexin. Changes in frequency of lysed and intact cells due to the antibiotic action uncovered several strains with atypical lysis kinetics. Filamentation confers tolerance because antibiotic removal before lysis leads to recovery through numerous concurrent divisions of filamented cells. Filamentation-mediated tolerance was not associated with resistance, and therefore this phenotype is not discernible through most antibiotic susceptibility methods. We find that deletion of Tol-Pal proteins TolQ, TolR, or Pal but not TolA, TolB, or CpoB leads to rapid killing by β-lactams. We also show that the timing of cell wall degradation determines the lysis and killing kinetics after β-lactam treatment. Altogether, this study uncovers numerous genetic determinants of hitherto unappreciated filamentation-mediated β-lactam tolerance and support the growing call for considering antibiotic tolerance in clinical evaluation of pathogens. More generally, the microscopy screening methodology described here can easily be adapted to study lysis in large numbers of strains.

## Introduction

Antibiotic tolerance is characterized by a slow killing rate of a bacterial cell population exposed to lethal concentrations of bactericidal drugs (Brauner et al., 2016). Clinical analysis of pathogens today mostly focusses on detecting the presence of antibiotic resistance whereas tolerance is rarely taken into account. Antibiotic resistance is quantified by the minimum concentration of a drug at which the pathogen fails to grow from an initial inoculum of approximately 10^5^ cells/ml (Wiegand et al., 2008). But the endpoint observation of no growth in minimum inhibitory concentration (MIC) tests does not guarantee that the antibiotic will exert sufficient bactericidal activity and will be successful in clearing an already established infection (10^8^–10^10^ cells/g of tissue or pus) (Levison and Levison, 2009). Also, measuring the MIC does not provide information regarding the time needed to kill the infectious agent. Killing rate is important as the antibiotic concentration does not stay constant in the human body like *in vitro* experimental conditions. Therefore, a therapeutic choice solely based on MIC values may fail because of an insufficient rate of killing. This is especially true for the class of β-lactam antibiotics because they exhibit little concentration-dependent killing and instead show time-dependent antibacterial activity (Turnidge, 1998). The duration of time for which the antibiotic level exceeds the MIC has been identified as the most suitable parameter to measure the bactericidal efficacy of β-lactam antibiotics (Vogelman et al., 1988; Turnidge, 1998). Not surprisingly, mounting evidence from studies in animal models and data from clinical isolates of bacteria support that tolerance to β-lactam antibiotics can cause relapse of bacterial infections and failure of antibiotic treatments (Rajashekaraiah et al., 1980; Handwerger and Tomasz, 1985; Grahn et al., 1987; Kim, 1988; Van den Bergh et al., 2016). The problem of antibiotic tolerance is not limited to complicating the eradication of pathogens by antimicrobial therapy. A recent study found that antibiotic tolerance increases the chances of acquiring resistance (Levin-Reisman et al., 2017). Therefore, antibiotic tolerance should be a grave concern and inequalities in the time needed to kill bacterial strains should be considered before a β-lactam treatment.

The molecular targets of β-lactam antibiotics are the penicillin-binding proteins (PBPs) which are crucial for cell wall synthesis in bacteria (Park and Strominger, 1957; Tipper and Strominger, 1965). It is now widely accepted that these bactericidal antibiotics cause cell lysis by creating an imbalance between the activities of the PBPs and the cell wall lytic enzymes (Tomasz, 1979). Physiological or phenotypic tolerance to β-lactam antibiotics was described in Hobby et al. (1942). Hobby et al. (1942) observed that density of cell culture and the growth rate affected killing kinetics of β-lactams. Genotypic tolerance was recognized nearly 5 decades ago in a pneumococci mutant with defective cell wall lytic enzymes (Tomasz et al., 1970). Upon incubation with penicillin, this mutant stopped growing but showed very little lysis and slower loss of viability. Since then many genetic, physiological and environmental factors have been implicated in β-lactam induced lysis and tolerance. Cell division plays a central role in lysis. Rapid lysis requires the successful assembly of the divisome machinery (Chung et al., 2009). Moreover, the rate of lysis is proportional to the cell generation time (Tuomanen et al., 1986; Lee et al., 2018). Lysis can be delayed or prevented in hypertonic medium or in presence of divalent cations (Joseleau-Petit et al., 2007; Yao et al., 2012). Protection of *E. coli* against lysis by lowering the pH of growth media also decreases the killing rate (Goodell et al., 1976). Amidases are cell wall lytic enzymes in *E. coli* that play a crucial role in daughter cell separation and β-lactam induced lysis (Heidrich et al., 2001). Simultaneous inactivation of two or three amidases delays the lysis (Chung et al., 2009).

Despite the fact that β-lactam induced killing usually culminates with lysis, systematic study of genetic factors affecting β-lactam lysis is still lacking. Most of the genome-wide antibiotic susceptibility screens performed using bacterial genetic libraries have centered on measurement of fitness (growth) on nutrient agar containing sub-MIC concentrations of antibiotics. For example, Nichols et al. (2011) measured the fitness of *E. coli* mutants in the Keio library (Baba et al., 2006) across several conditions (including sub-MIC concentrations of different β-lactams). Although this approach has yielded numerous valuable insights, fitness in presence of sub-MIC concentrations of a drug cannot simply be correlated to killing kinetics in presence of bactericidal concentrations.

Penicillin-binding proteins 3, also known as FtsI, is an essential cell division protein in *E. coli* and is required for septal cell wall synthesis (Spratt, 1975; Typas et al., 2012). Many β-lactams such as cephalexin, aztreonam, ceftazidime, and piperacillin show strong affinity to PBP3 in *E. coli* (Spratt, 1975; Hayes and Orr, 1983; Hakenbeck et al., 1987; Rittenbury, 1990; Kocaoglu and Carlson, 2015). These antibiotics cause lysis albeit after a period of cell filamentation (Spratt, 1975; Chung et al., 2009). Antibiotic removal or revival of PBP3 activity before lysis leads to synchronous divisions in filamented cells (Botta and Park, 1981). Therefore, duration of the cell filamentation influences killing kinetics and filamentation-mediated delay in lysis can potentially confer antibiotic tolerance. Enabled by the fact that cell lysis can be observed with phase contrast microscopy, we used high-throughput time-resolved microscopy to measure cell lysis kinetics of all mutants in the single non-essential gene deletion library of *E. coli* (Baba et al., 2006) in response to the β-lactam antibiotic cephalexin. The automated microscopy screen and image analysis revealed several genes that play a role in the onset of lysis. Delayed lysis confers antibiotic tolerance because the filamentous cells resulting from antibiotic exposure can recover by undergoing concurrent divisions after the antibiotic removal. Standard antibiotic susceptibility measurements did not yield any difference between the wild type (*E. coli* BW25113) and the most tolerant strains, highlighting the novelty of the methodology, and the phenotypes discovered. The genetic determinants of filamentation-mediated tolerance to β-lactams unraveled in this study will be highly instrumental in uncovering new mechanisms of antibiotic tolerance.

## Results and Discussion

### Microscopy-Based Quantification of β-Lactam Lysis Kinetics

Time-resolved determination of the number of viable cells surviving antibiotic treatment provides information about the killing kinetics of the antibiotic. The gold standard method employed for determining viable cell count is counting of colony forming units (CFU) on nutrient plates. CFU counting is very accurate but slow and labor-intensive and is therefore not suitable for high-throughput experiments. The action of β-lactam antibiotics can also be observed in the optical density (OD) measurements because cell lysis causes a decrease in OD. OD readings can be taken automatically in a high-throughput manner using a microplate reader, but can be converted to cell counts only if a calibration curve is available (Stevenson et al., 2016). Since β-lactam induced filamentation can lead to a wide variety of cell lengths, skewing the correlation between OD and cell counts, it is not possible to accurately use OD readings for measuring lysis kinetics for large number of strains. Another shortcoming of using OD to measure lysis is related to the dependence of lysis to the generation time (Tuomanen et al., 1986). The steady state growth of *E. coli* stops when cell culture reaches the OD of 0.3 (Sezonov et al., 2007) and then the growth rate slows down abruptly. Cell lysis kinetics should thus be measured when cell densities are much lower than the density corresponding to OD = 0.3, but at those cell densities OD may not be measured reliably.

**FIGURE 1:**
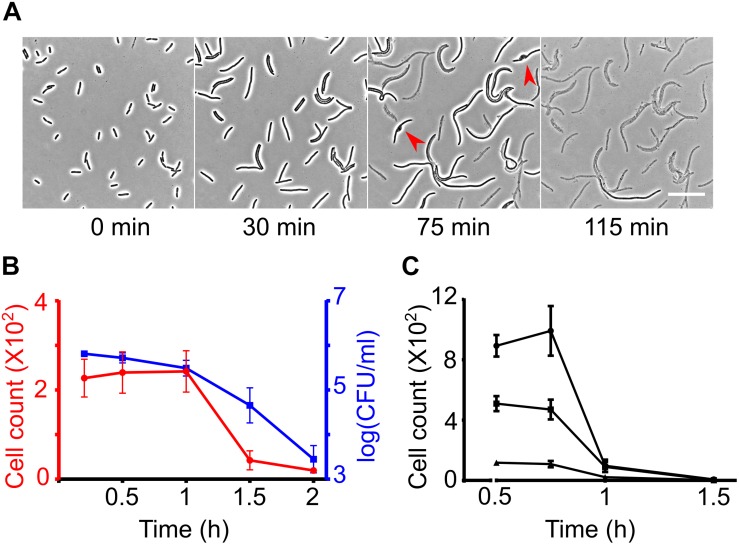
Measuring β-lactam induced lysis kinetics by phase contrast microscopy. **(A)** Time-lapse analysis of wild-type cells on agar pads containing cephalexin 100 μg/ml. Cells immediately stop dividing after seeded on the cephalexin containing agar but continue to elongate. After growing to variable lengths, the cells develop a bulge (shown by red arrow heads) and lyse. Scale bar corresponds to 25 μm. **(B)** Graph shows wild-type *E. coli* cell counts at different time intervals after treatment with cephalexin 100 μg/ml at *t* = 0 in glass-bottom imaging microplates. Cell counts were determined simultaneously by image analysis (left axis in red) and by CFU counting on agar plates (right axis in blue). **(C)** Wild-type *E. coli* was treated with cephalexin 200 μg/ml in glass-bottom 96-well plates at different cell densities and cell counts were determined based on image analysis. All data points correspond to 3 replicates in **(B,C)**.

Recently, we developed a method for high-throughput time-resolved imaging of bacteria in 96-well plates (Zahir et al., 2019). This method is ideal for recording dynamic single-cell responses of a large number of strains in the form of high-resolution phase contrast images. It utilizes a custom developed image acquisition routine to image strains arrayed in 96 wells for multiple time-points in an automated way without the need to manually define the spatial location of 96 strains or the position of the focal plane. The method also employs real-time analysis of images and updates the hardware settings to compensate for changes in cell number and cell morphology over time. The methodology is promising for measuring β-lactam induced lysis kinetics as lysed cells can be easily distinguished from intact cells in phase contrast microscopy images which enable viable cell counting. Moreover, our methodology allows us to work with low cell densities (10^5^ – 10^7^ cells per ml); which is ideal, because the growth rate remains robust to changes in cell density during filamentation and also the variability in cell density across mutants does not affect the assay. Our goal was to establish a microscopy screen that would be capable of identifying both the rapidly lysing and the late lysing strains in a library. To find the right conditions for the screening, we first monitored the response of *E. coli* to three β-lactams that are PBP3 inhibitors – cephalexin, aztreonam and piperacillin. Cephalexin showed the most promise among these antibiotics. Like other β-lactams, the timing of the onset of lysis depends upon the concentration of cephalexin (Supplementary Figure S1). Treatment of *E. coli* with more than 100 μg/ml cephalexin causes cells to start lysing within 1 h (Figure 1A). In case of aztreonam and piperacillin, at various concentrations we found that cells filament for hours before lysing. Extensive filamentation would negatively affect the sensitivity of the microscopy screen because many mutants would filament for hours and enter the stationary phase without lysing, preventing any further differentiation. Next, we checked if the microscopy screening methodology can be applied to measure lysis kinetics reliably despite the filamentation. We simultaneously obtained CFU counts and recorded images of bacterial cultures in a glass-bottom 96-well plate after treatment with cephalexin 100 μg/ml. This concentration of cephalexin induces extensive filamentation (Figure 1A). The CFU counts decreased slowly till 1 h and then decreased very sharply (Figure 1B). The automated counts from the microscopy images also showed a steep decline after 1 h (Figure 1B). Since the cell count is independent of the length of the cells, it did not show much increase after the antibiotic was added and then decreased sharply as the cells started lysing. Next, we assessed the suitability of the approach for implementation on a genomic library. More specifically, we checked the robustness of lysis kinetics to changes in cell density in our approach. The concentration of 25 × MIC (200 μg/ml) was chosen for the genome-wide screen because it provided us enough time to take 1 set of images per mutant before the wild type starts lysing. The images from this time-point can thus reveal rapidly lysing mutants. Using this concentration of the antibiotic also provided us plenty of time after lysis of the wild type (around 1 h after treatment) to monitor the response of late lysing mutants before they reach stationary phase. Intact cell counts from wells treated with the antibiotic at different growth phases showed that the onset of lysis was robust to varying cell density (Figure 1C). The robustness can be attributed to the requirement of low cell densities for this methodology. At low cell densities, the growth rate and thereby the lysis kinetics stay robust to changes in growth phase or the cell count. These results together suggest that high-throughput time-resolved microscopy can be used for large-scale phenotyping to identify strains or conditions that exhibit atypical lysis kinetics.

### High-Throughput Microscopy Screening for Atypical Lysis

**FIGURE 2:**
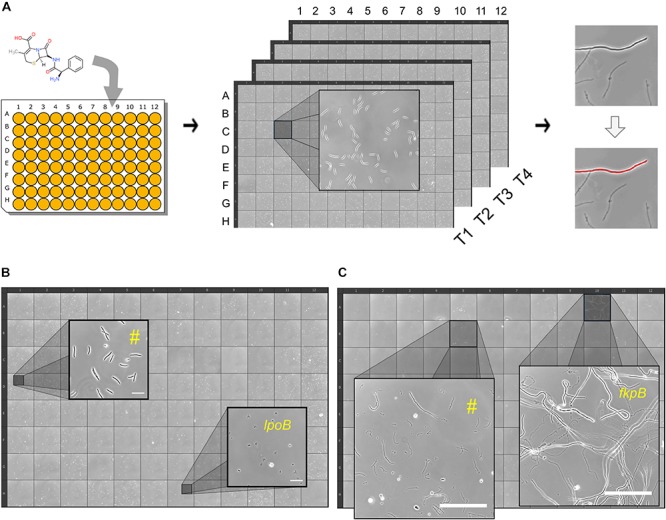
High-throughput time-resolved microscopy screen for atypical lysis kinetics. **(A)** Shown is a simplified schematic of the workflow. Cephalexin is added to growing cell cultures in glass-bottom 96-well plates and then the plate is kept on the microscope. Images are taken at 4 time-points: 40 min, 85 min, 2 and 4.5 h. Images are then processed to count the number of intact cells at every time-point for each well/strain. Lysed and intact cells are distinguished by thresholding. **(B)** 96-well plate view of an experiment at T_40__*min*_. Zoomed insets show two wells with different phenotypes. Well marked with (^#^) is representative of most strains, showing intact elongated cells. Well containing the rapidly lysing strain Δ*lpoB* shows lysed cells at T_40__*min*_. Scale bars correspond to 20 μm. **(C)** 96-well plate view of an experiment at T_4_._5h_. Zoomed insets show two wells with different phenotypes. Well marked with (^#^) is representative of most strains, showing lysed cells. Well containing the late lysing strain Δ*fkpB* with intact cells. Scale bars correspond to 100 μm.

We developed an image-based screen to find *E. coli* non-essential gene deletion mutants that display rapid or late onset of lysis to cephalexin (Figure 2A and section “Materials and Methods”). Briefly, strains from the Keio library (Baba et al., 2006) were grown to exponential phase (around 10^6^ CFU/ml) in glass bottom 96-well plates. Cephalexin was added to a final concentration of 200 μg/ml (25 × MIC of the wild type) in each well and then the responses of the mutants were recorded in the form of images at 4 different time-points. These time-points correspond to 32–48 min (T_40__*min*_), 76–92 min (T_85 *min*_), 116–132 min (T_2h_), and 265–282 min (T_4_._5h_) after antibiotic treatment. To provide a reference, 96 replicates of the wild type were also grown, treated with cephalexin and imaged under the same conditions as the mutants (Supplementary Figure S2). In addition, as a quality control, we kept the wild type in one well for every experiment. Before analyzing the image data, we verified previously known phenotypes. At T_40 *min*_ wild-type cultures only showed filamented cells. We speculated that if there is early onset of lysis in a mutant, we would see lysed cells in images from T_40 *min*_. Consistent with our hypothesis and in agreement with previous reports, we found that the wells containing *slt* (Templin et al., 1992), *mrcB*, and *lpoB* (Typas et al., 2010) deletion strains had a large number of lysed and bulging cells. Figure 2B shows a 96-well view of T_40 *min*_ from one experiment with a zoomed inset showing lysed Δ*lpoB* cells. We then looked for obvious late lysis phenotypes in the images from T_4_._5h_. The Δ*fkpB* strain stood out from the rest (Figure 2C). While all strains (including the wild type) showed mostly lysed cells, Δ*fkpB* showed predominantly intact cells up to 200 μm long at T_4_._5h_.

**FIGURE 3:**
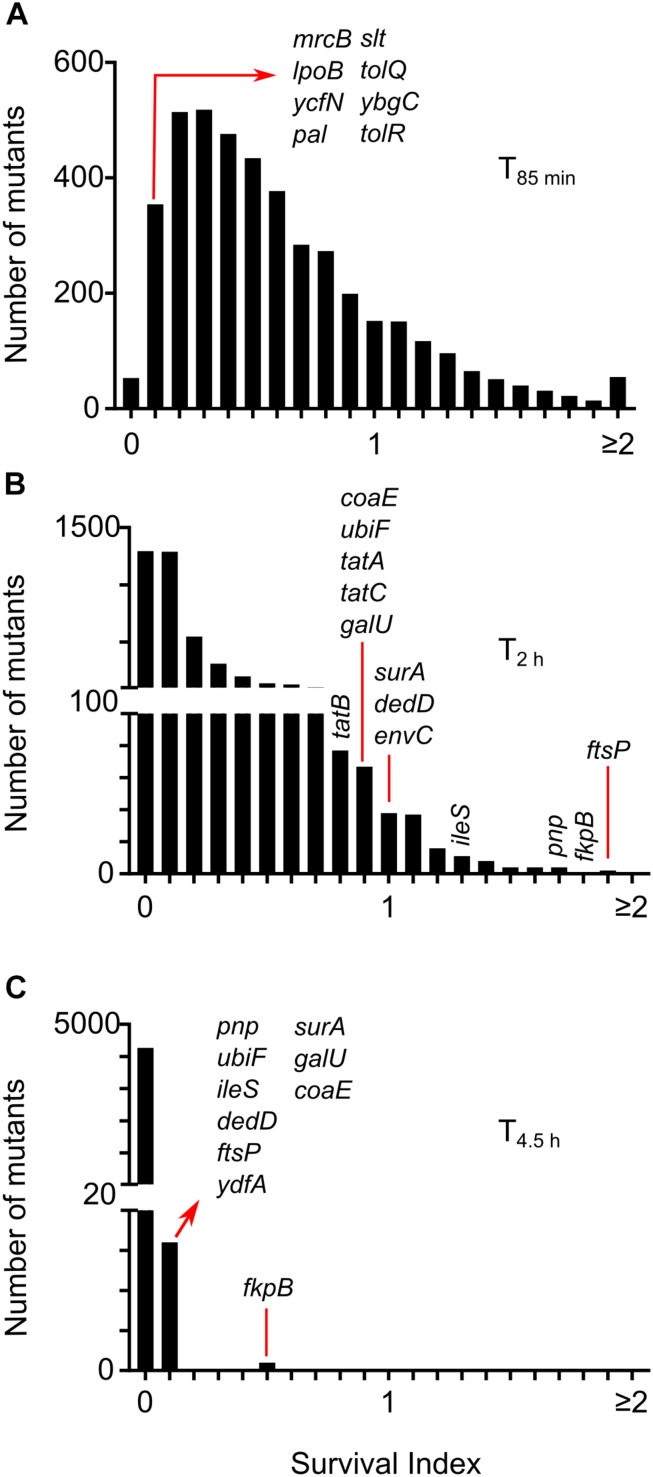
Lysis kinetics of *E. coli* single gene deletion mutants following treatment with cephalexin. Distribution of survival index (SI) for all the strains at time-points T_85__*min*_ **(A)**, T_2__h_ **(B)**, and T_4_._5h_ **(C)**. SI is calculated by taking the ratio of intact cells at each time-point to the number of intact cells segmented at a previous time-point (T_40__*min*_ or T_85__*min*_; whichever is more). Positions of genes discussed in the text are shown.

Images were processed to count the number of intact or non-lysed cells at four time-points for 4320 strains (Supplementary Data Sheet S2) and the wild-type replicates (Supplementary Data Sheet S2). We estimated lysis kinetics by computing a parameter referred to as the survival index (SI) from here onward in the text. SI is the ratio of intact cells at a certain time-point and the number of intact cells segmented at a previous time-point (T_40__*min*_ or T_85__*min*_; whichever is more). SI of all wild-type wells in the reference experiment was below 1 at T_85__*min*_ (Supplementary Figure S2), indicating that the wild type starts lysing before 85 min in the conditions specific to the screen. SI at T_85__min_ for 3546 strains was below 1 (Figure 3A) indicating that most of the strains in the library display lysis kinetics similar to the wild type. At T_2h_ wild-type cells predominantly lyse (Supplementary Figure S2). Remarkably, we found that 102 strains showed SI > 1 at T_2__h_, indicating a considerable delay in the onset of lysis (Figure 3B). At T_4_._5h_ most of the wells (3256 mutants) had zero intact cells and only Δ*fkpB* showed SI higher than 0.1 (Figure 3C). We also manually inspected the images of 667 strains which showed an SI lower than 0.2 at T_85__*min*_ to identify the strains that already started lysing at T_40__min_ and found 8 mutant wells – Δ*mrcB*,Δ*lpoB*,Δ*slt*,Δ*ybgC*,Δ*tolQ*,Δ*tolR*,Δ*pal*, and Δ*ycfN* that contained lysed cells at T_40__*min*_ (Figure 3A).

The high-throughput time-resolved microscopy methodology enabled us to enumerate the living cells following β-lactam treatment at low cell densities, in exponentially growing cultures. Working with low cell densities also proved to be of paramount importance to the success of finding extreme tolerance phenotypes (like the Δ*fkpB* strain) as none of the strains experienced growth saturation before onset of the lysis. Altogether, the screen identified several undescribed phenotypes, and it uncovered the extensive impact of the genotype on β-lactam lysis kinetics. Several single non-essential gene deletions can delay lysis. This observation is alarming and suggests that the prevalence of antibiotic tolerance in clinical strains could be underexplored. An exciting aspect of the high-throughput microscopy screening method described here is the potential for extending its use to other agents causing lysis and filamentation. Several groups of antimicrobials cause cell lysis like β-lactams, antimicrobial peptides, and glycopeptides (Kohanski et al., 2010). Bacterial filamentation can be caused by a variety of stresses like antibiotics (Domadia et al., 2007), DNA damage (Kantor and Deering, 1966), host immune responses (Möller et al., 2012), and temperature (Jones et al., 2004). This methodology is ideal for long term monitoring of bacterial shape changes and should be readily adaptable to other organisms and enable similar high-throughput screens to discover genetic determinants of processes that involve lysis or filamentation of bacteria.

### Deletion of TolQ, TolR, or Pal Causes Rapid Lysis

The presence of lysed cells in images taken at T_40*min*_ indicated early onset of lysis in the following 8 mutants – Δ*mrcB(PBP1B)*, Δ*lpoB*, Δ*slt*, Δ*ybgC*,Δ*tolQ*, Δ*tolR*, Δ*pa*, and Δ*ycfN*. Deletion of either *mrcB* or *lpoB* causes hypersensitivity to various β-lactams (Denome et al., 1999; Paradis-Bleau et al., 2010). The soluble lytic transglycosylase (*slt*) mutant of *E. coli* is known to form bulges and lyse rapidly when PBP3 is inhibited (Imada et al., 1982; Templin et al., 1992). TolQ, TolR, and Pal are part of the Tol-Pal complex which is crucial for proper membrane invagination during cell division (Gerding et al., 2007). The position of the kanamycin cassette was confirmed by PCR. The kanamycin cassette from these strains was re-introduced into the wild type and then the cassette was excised (see section “Materials and Methods” for details). All strains still retained their rapid lysis phenotype. *ybgC* lies upstream in the same operon as *tolQ* and *tolR* and the *ycfN* gene overlaps with *lpoB*. Considering this, we additionally performed complementary assays for Δ*ybgC* and Δ*ycfN*. As a control, first we tried to see if we can complement Δ*mrcB* and revert the cephalexin response to the wild type. Expression of *mrcB* in *trans* from the pBAD plasmid eliminated the early lysis phenotype of Δ*mrcB* (Supplementary Figure S3A), whereas the empty pBAD vector didn’t change the phenotype. Next, we cloned *lpoB* into the pBAD and expressed it in Δ*ycfN* cells. Expression of *lpoB* eliminated the rapid lysis phenotype in Δ*ycfN* (Supplementary Figure S3B) confirming that the rapid lysis phenotype is due to deletion of overlap region of *lpoB* gene. Also, *trans* expression of *ybgC* could not revert the rapid lysis phenotype in Δ*ybgC* (Supplementary Figure S3C) indicating that that the phenotype is due to polar effects of the *ybgC* deletion on the downstream *tolQ/R* genes. Hence, we did not consider Δ*ycfN* and Δ*ybgC* for further experiments.

**FIGURE 4:**
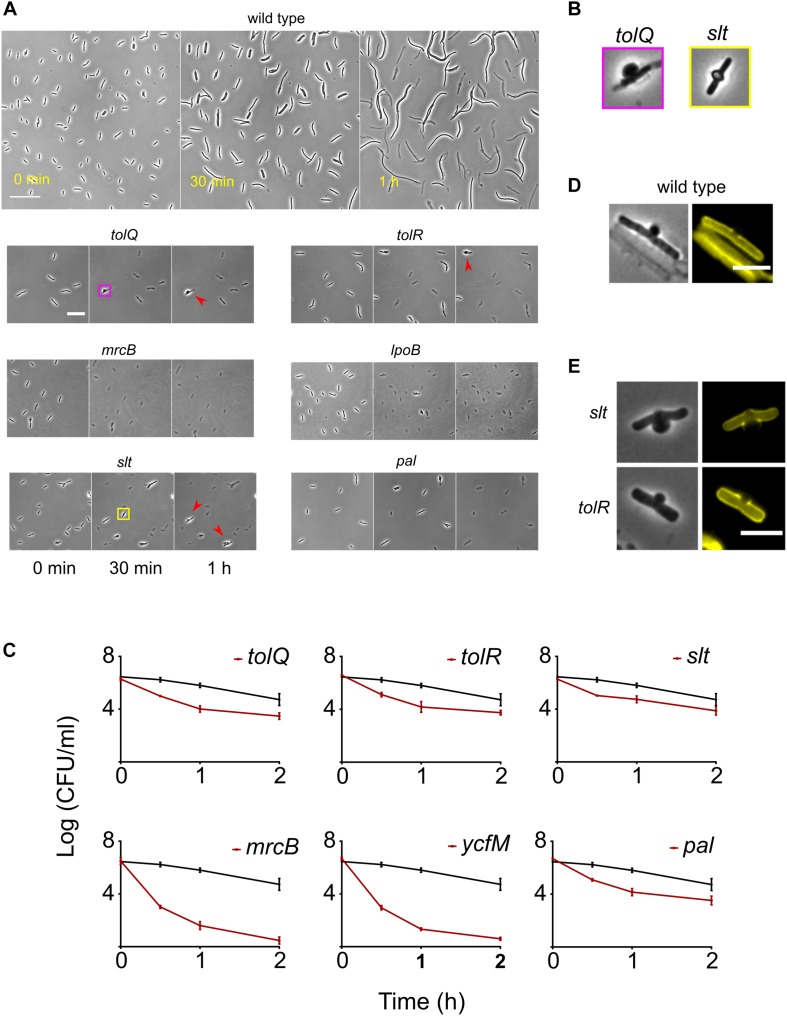
Response of the wild type and the rapidly lysing strains to cephalexin. **(A)** Shown are the micrographs of the wild type and the rapidly lysing strains after seeding on agar pads containing 50 μg/ml of cephalexin. The wild type elongates and starts to lyse only after 30 min whereas all the mutants start lysing immediately. After 1 h, a small fraction of Δ*tolQ*, Δ*tolR*, and Δ*slt* cells stay intact but their morphology is grossly altered. These cells display stable large bulges (shown by red arrowheads). The yellow and pink square insets are enlarged in **(B)**. **(B)** The bulge morphology of Δ*slt* is different from Δ*tolQ* and Δ*tolR*. Bulges in Δ*slt* protrude on both sides whereas Δ*tolQ* and Δ*tolR* cells have protrusion only on one side. **(C)** Time-kill curves of cephalexin against all rapidly lysing strains and the wild type is shown. Cephalexin 50 μg/ml was added at time 0 h. The wild type is shown by a black curve in all plots. Due to cell lysis, the number of surviving cells declines very quickly for all mutants. *mrcB* and *lpoB* deletion strains exhibit the fastest decline in viable cells. All data points correspond to 3 replicates. Phase contrast and fluorescence images of bulging wild-type **(D)**, Δ*slt* and Δ*tolR* **(E)** cells. Fluorescent images show cell walls stained with WGA-Tetramethylrhodamine. The wild type was imaged 25 min after adding cephalexin, while Δ*slt* and Δ*tolR* cells were imaged after 10 min. In the image of the wild type we can see debris from lysed cells and one intact cell with a bulge. In the wild type and Δ*tolR* cells, the bulge protrudes on one side. Their cell wall is also fractured only on one side, whereas Δ*slt* cell has protrusion and cell wall damage on both sides (albeit unequal in severity). Scale bar corresponds to 30 μm in **(D)** and 20 μm in **(E)**.

We monitored the response of rapidly lysing mutants on agar pads containing 50 μg/ml (instead of 200 μg/ml used in the high-throughput microscopy assay) of cephalexin by time-lapse microscopy. The concentration of 50 μg/ml was chosen for slower lysis kinetics and better separation in time between the onset of lysis of the wild type and rapidly lysing strains. Much to our surprise, even at 50 μg/ml all of the rapidly lysing strains started bulging and lysing immediately at the first round of cell division, while the wild type first elongated and then lysed (Figure 4A). Early onset of lysis was also observed with another PBP3 specific β-lactam ceftazidime (Supplementary Figure S4). Time-lapse microscopy revealed different patterns of cell lysis. All mutants showed cells with bulges at mid-point position before lysis, but the duration of bulging was different. In the case of *mrcB*, *lpoB*, and *pal* deletion strains bulges were extremely short-lived and thus cells with bulges were rarely observed. In case of *slt*, *tolQ*, and *tolR* deletion strains, a small fraction of cells formed very stable bulges (Figure 4A: red arrowheads). The morphology of bulges also differed between the mutants. Δ*tolQ* and Δ*tolR* mutants showed a unilateral protrusion from the cell surface while the mutant lacking the *slt* gene showed characteristic localized swelling (Imada et al., 1982) which encircled the entire circumference of the rod (Figure 4B). Concurrent to our observations in time-lapse microscopy assay, time-kill curves of the rapidly lysing strains showed a rapid decay in viable cell counts when treated with cephalexin 50 μg/ml (Figure 4C). Since β-lactams inhibit the cell wall formation, we suspected that the rapidly lysing strains would show cell wall damage immediately after exposure to cephalexin. We examined the cell wall morphology of the wild type, Δ*tolQ* and Δ*slt* strains after exposure to cephalexin. Wild-type cells start showing bulges and cell wall fractures albeit after 30 min of filamentation (Figure 4D and red arrows in Figure 1A), whereas Δ*tolQ* and Δ*slt* showed membrane bulges and a fractured cell wall only after 10 min (Figure 4E). In agreement with their morphology of bulges, Δ*slt* showed a cleft in the cell wall on both sides while Δ*tolQ* displayed a one-sided fracture. Deletion of *tol-pal* genes affects outer membrane integrity in *E. coli* and increase the permeability of the membrane (Bernadac et al., 1998; Cascales et al., 2002; Gerding et al., 2007). Therefore, rapid lysis of *tolQ*, *tolR*, and *pal* could be caused by increased intake of cephalexin, but we found that deletion of other Tol-Pal complex members *tolA*, *tolB*, and *cpoB* does not change lysis kinetics even though they also show cell envelope defects and increased permeability (Kowata et al., 2016). This observation points that rapid lysis is not due to increased intake of cephalexin. Besides, a study of β-lactam permeability found that *E. coli* is highly permeable to cephalexin (Sawai et al., 1979). The sensitivity of *tol-pal* mutants to various detergents and cell wall antibiotics like vancomycin and β-lactams are widely reported (Lazzaroni et al., 1999; Nichols et al., 2011). Nichols et al. (2011) showed that *tol-pal* mutants are less fit to grow on nutrient agar containing sub-inhibitory concentration of β-lactam antibiotics. Although, it is tempting to assume rapid killing of *tol-pal* mutants that is described here from this previous chemical genomics study, caution must be exercised. Mutants like Δ*tolB*, Δ*cysB*, and Δ*fis* which show lysis kinetics similar as the wild type are actually more sensitive to several β-lactams (including ceftazidime) than the rapidly lysing *tol-pal* mutants in the conditions described for the chemical genomic screen (Nichols et al., 2011). Moreover, mutants like Δ*tatB* and Δ*ompA* that show extensive filamentation were instead also found to be sensitive to ceftazidime in the same study.

Quick and efficient eradication of pathogens is important to avoid recurrence of infection, particularly in immune-compromised patients. For this reason, it is preferable that β-lactam treatment leads to rapid lysis of pathogens. β-lactam antibiotics that preferentially bind to PBP3 cause cells to lyse, albeit after a period of cell elongation. Peters et al. (2011) reported a fail-safe mechanism that ensures amidase mediated cell wall hydrolysis does not start in *E. coli* until the septal cell wall synthesis machinery is ready. In β-lactam induced filamented cells the amidases fail to localize to the septal rings (Peters et al., 2011) and thus the fail-safe mechanism prevents cell wall hydrolysis (and lysis) when the cell wall synthesis is inhibited. We found that Δ*tolQ*, Δ*tolR*, and Δ*pal* lyse rapidly after β-lactam treatment. Combining β-lactams with inhibitors of these proteins should result in faster killing of the pathogens at lower concentration of antibiotic. We argue that rapid lysis of these *tol-pal* deletion strains is not a manifestation of their compromised cell envelope or increased membrane permeability because of two reasons. Δ*tolA*, Δ*tolB*, and Δ*cpoB* exhibit cell envelope defects (Gerding et al., 2007; Gray et al., 2015) and membrane permeability (Kowata et al., 2016) very similar to Δ*tolQ*, Δ*tolR*, and Δ*pal* but they do not lyse rapidly in presence of cephalexin. Secondly, cephalosporins (including cephalexin) have high permeability into *E. coli* cells without any cell envelope defects (Sawai et al., 1979). Although the Tol-Pal complex serves a major role in maintaining outer membrane integrity during cell division (Bernadac et al., 1998; Gerding et al., 2007), the inability of the *tol-pal* deletion strains to stop cell wall degradation following PBP3 inhibition described here seems to be due to breakdown of the fail-safe mechanism reported by Peters et al. (2011). Indeed, recent publications support the conjecture of a crucial role of Tol-Pal proteins in cell wall remodeling during division (Typas et al., 2010; Gray et al., 2015; Tsang et al., 2017).

### Delay in Lysis Confers Tolerance but Not Resistance

**FIGURE 5:**
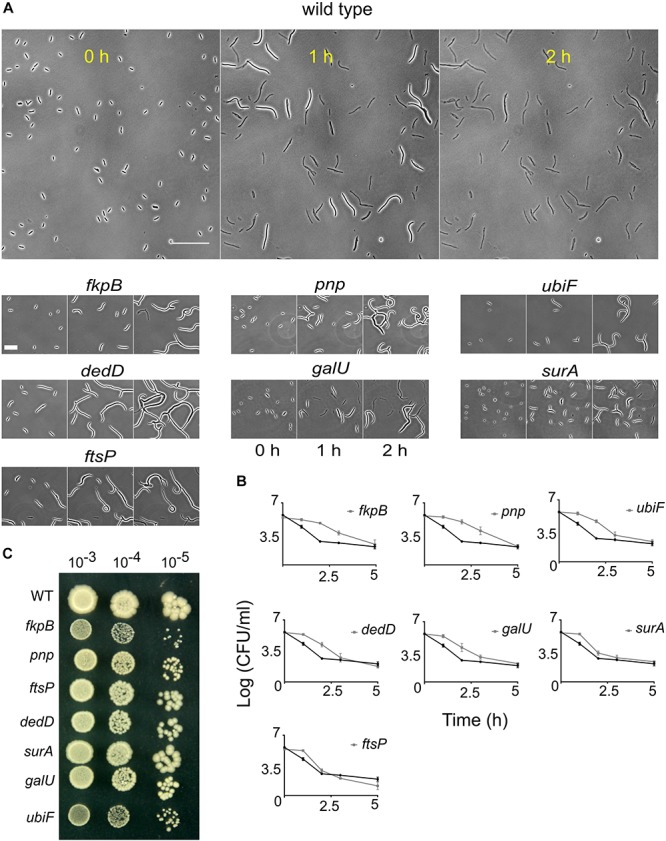
Response of the wild type and the late lysing strains to cephalexin. **(A)** Shown are the micrographs of the wild type and 10 late lysing strains after seeding on agar pads containing 200 μg/ml of cephalexin taken at 0, 1, and 2 h after seeding on cephalexin-containing agarose. The wild type starts to lyse before 1 h and after 2 h no intact cells are left. Scale bar on the image of the wild type corresponds to 50 μm. In contrast to the wild type, late lysing strains show considerable numbers of intact cells even after 2 h of treatment. Scale bar for mutant micrographs corresponds to 25 μm. **(B)** Shown are time-kill curves of cephalexin against the 10 late lysing strains and the wild type. Cephalexin 200 μg/ml was added at time 0 h. The wild type is shown by the black curve in all plots. All mutant strains (gray curves) show increased survival compared to the wild type after 2 h. All data points show the mean and standard deviation of 3 replicates. **(C)** Spot titer assay of the wild type (WT) and the tolerant strains is shown. Strains were plated on LB agar media containing cephalexin 2 μg/ml.

From the microscopy screen, we identified tolerant strains that filamented for longer duration than the wild type. To characterize the phenotype of filamentation-mediated late lysis, we selected the 10 strains (Δ*fkpB*, Δ*pnp*, Δ*ubiF*, Δ*ileS*, Δ*dedD*, Δ*ydfA*, Δ*coaE*, Δ*ftsP*, Δ*galU*, and Δ*surA*) showing the highest values of SI at T_4_._5h_ (Figure 3C and Supplementary Data Sheet S1). Again, the kanamycin cassette from these strains was re-introduced into the wild type and then the cassette was excised. We also confirmed the position of kanamycin cassette by PCR. After excision of the kanamycin cassette, the strains Δ*ileS*, Δ*ydfA*, and Δ*coaE* lost their filamentation phenotype. Indeed, Δ*coaE* strain in the Keio library has a duplication (Yamamoto et al., 2009) so the filamentation phenotype is not due to the absence of CoaE. The β-lactam induced filamentation phenotype in these strains could instead be a result of overexpression of genes downstream of the kanamycin promoter. We took the rest of the 7 mutants for further experiments. The response of these strains to cephalexin was investigated in more detail by time-lapse microscopy of cells seeded on agarose pads containing cephalexin (200 μg/ml). While the wild-type lyses completely in 2 h, these strains keep filamenting and show intact cells (Figure 5A). Slower growth leads to slower lysis. So, we measured the generation time of these strains at the growth phase used in the screen. Only Δ*pnp* and Δ*ubiF* multiplies slower than the wild type (Supplementary Table S1). But, a higher generation time still does not explain how Δ*pnp* cells can avoid lysis and grow up to 58.2 ± 12.5 μm (*n* = 17) in 2 h while the wild type cells lyse when they are only 29 ± 6.3 μm (*n* = 46) long. These strains also showed filamentation phenotype with ceftazidime (Supplementary Figure S5). We then measured the killing kinetics of these strains by CFU counting on LB agar plates. For all the strains we observed a higher survival rate compared to the wild type at least until 2 h after adding the antibiotic (Figure 5B). Time-kill assays showed distinct patterns of killing. For the wild type, Δ*ftsP* and Δ*surA* it takes 1–2 h to kill 99% of cells. In case of Δ*ubiF*, Δ*dedD*, and Δ*galU* it took 2–3 h to kill 99% of cells and for Δ*fkpB* and Δ*pnp* it took 3–5 h (Figure 5B). The plate counts (survival) after antibiotic treatment is dependent not only on the ability of cells to evade lysis but also their ability to recover and form a colony after the antibiotic is removed. Although microscopy analysis showed that these strains predominantly do not lyse in the first 2 h, reduction in cell viability could still be due to lysis during recovery. Next, we checked whether the filamentation phenotype can be discerned using other traditional antibiotic susceptibility assays. The MIC and minimal bactericidal concentration (MBC) values of these strains are the same as those of the wild type (Supplementary Table S2). Spot titer assay for cephalexin (2 μg/ml; MIC/4) sensitivity did not show any difference in sensitivity between the tolerant strains and the wild type (Figure 5C). These results together show that filamentation phenotype confers tolerance but not resistance and most antibiotic susceptibility assays are not suitable for identification of filamentation mediated tolerance.

**FIGURE 6:**
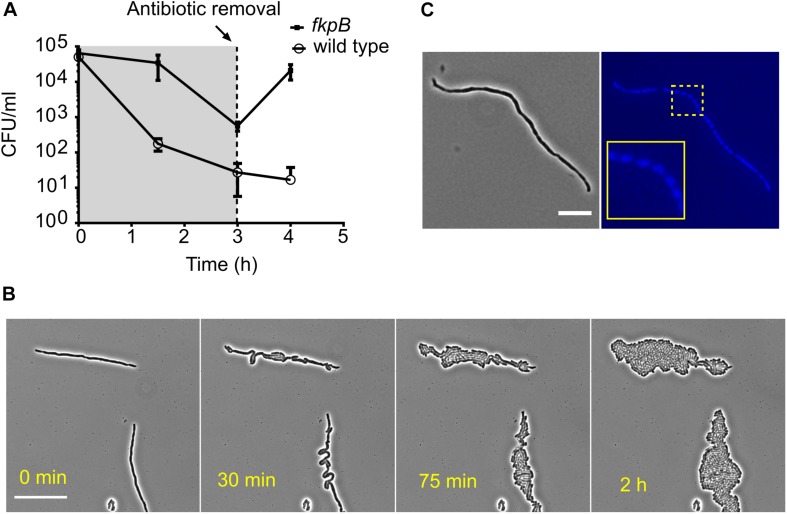
Filamented cells arising from prolonged β-lactam exposure are multinucleated and revert to normal growth once the antibiotic is removed. **(A)** Shown are the time-kill curves of the wild type and the tolerant strain Δ*fkpB* in the presence of cephalexin (200 μg/ml). The antibiotic was washed away after 3 h of exposure and fresh media was added to allow regrowth of the remaining cells. Antibiotic removal leads to explosive multiplication in case of Δ*fkpB*. All data points correspond to 3 replicates. **(B)** Shown is the recovery of filamented Δ*fkpB* cells on LB-agar pads after removal of the antibiotic. Cells were collected after 2 h of exposure to cephalexin (200 μg/ml). The explosive growth after antibiotic removal in **(A)** can be explained by multiple simultaneous divisions in the filamented cells. The scale bar corresponds to 25 μm. **(C)** A filamented Δ*fkpB* cell stained with the nucleic acid dye DAPI is shown in the micrograph. Segregated DAPI foci can be seen in the blue channel indicating the presence of multiple nucleoids and perpetuation of the chromosome replication process during elongation inside the cell. The scale bar corresponds to 10 μm.

The slow killing kinetics of severely filamenting strains in the time-kill assays indicate that at least some of the filamented cells were viable and capable of forming a colony on nutrient agar plates. The viability of Δ*fkpB* for example, does not undergo any substantial change after cephalexin treatment for 2 h, but the cells keep growing in mass. To better understand the implications of filamentation-mediated tolerance on post-antibiotic recovery, we examined the effect of antibiotic removal after a period of filamentation. We therefore treated the wild type and strain Δ*fkpB* with cephalexin and then removed the antibiotic after 3 h. When the antibiotic was removed, the CFU counts of the Δ*fkpB* strain increased more than 25-fold in the next hour (Figure 6A). This dramatic increase in cell counts can be attributed to separation of one long cell into multiple individual sister cells. Figure 6B shows how Δ*fkpB* cells can divide simultaneously to form a colony after antibiotic removal. DAPI (4’,6-diamidino-2-phenylindole) staining showed that filamented cells were multinucleated (Figure 6C). A similar response was also observed for the wild-type filamented cells after a 2-h exposure although to a much lower dose of 16 μg/ml cephalexin instead of the 200 μg/ml used in the high-throughput microscopy screen (Supplementary Figure S6). So, filamentation ensures transient growth in disguise even in bactericidal concentrations of β-lactam antibiotics.

Besides inducing lysis, other modes of action of β-lactams include lethal malfunctioning of cell wall assembly and accumulation of hydroxyl radicals (Kohanski et al., 2007; Cho et al., 2014). However, the extent to which these factors contribute to cell death is not clear. It has been shown that *E. coli* grown under osmoprotective conditions suffers from gross morphological changes under the influence of a β-lactam antibiotic. Nonetheless, these cells still start dividing into normal cells once the antibiotic pressure is removed (Yao et al., 2012). β-lactams are also not effective against pathogens such as *Vibrio cholera* and *Pseudomonas aeruginosa* because they can evade lysis by converting to spherical cells (Monahan et al., 2014; Dörr et al., 2015). These observations already suggest that lysis is responsible for killing the bulk of cells following β-lactam treatment. In this study, we further reinforce the idea that tolerance to β-lactams can arise due to slow lysis. Before lysis, there is a period of filamentation during which cells are viable. Even more so, the filamentation phase following antibiotic treatment creates multinucleated cells, which are essentially multiple cells waiting to be segregated once the antibiotic level falls. Since DNA replication continues inside these filamentous cells, they may facilitate the emergence of resistance in the population (Bos et al., 2015). Filamentation also promotes evasion of phagocytosis-mediated killing in hosts (Yang et al., 2016), which makes the scenario even more problematic. Interestingly, filamentation in uropathogenic *E. coli* is observed during urinary tract infections as a response to host innate immunity (Justice et al., 2006, 2008). This filamentation response could provide fortuitous tolerance to β-lactams.

The β-lactam-mediated lysis rate is linearly proportional to growth rate (Tuomanen et al., 1986; Lee et al., 2018), but surprisingly, the most tolerant strains discovered in our study have same generation time as the wild type. This suggests other mechanisms of β-lactam tolerance in these strains. We also found that antibiotic resistance is not a pre-requisite for phenotypic tolerance toward β-lactams. Therefore, tolerance cannot be discerned by standard antibiotic susceptibility tests that measure endpoint growth (like MIC and MBC measurements). Instead, we need to adopt approaches that look at dynamics of cell death for studying the mechanisms of antibiotic tolerance.

### Network Analysis of β-Lactam Tolerance Genes

**FIGURE 7:**
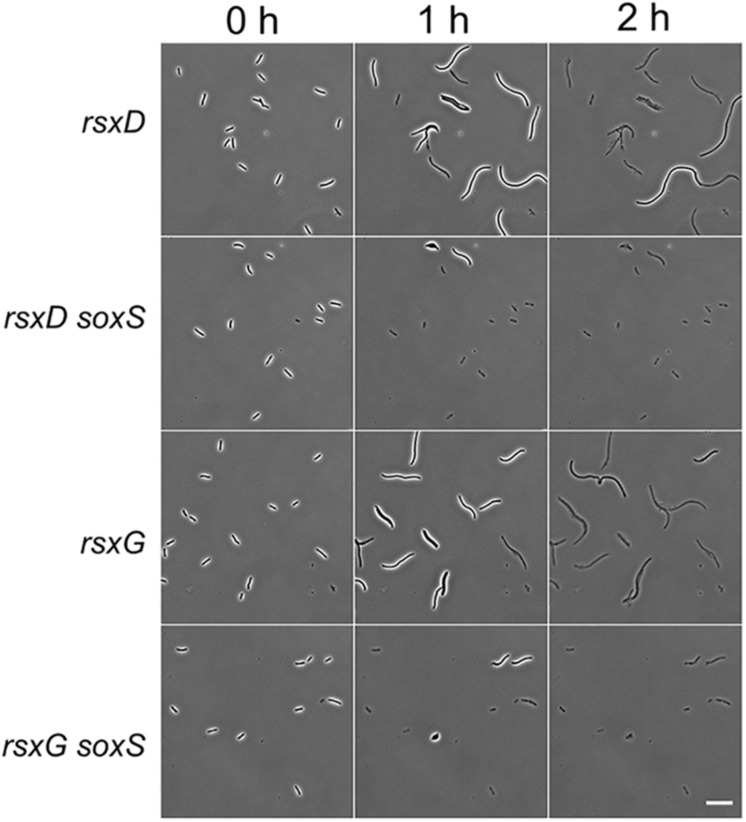
SoxS is necessary for the filamentation response in *rsx* deletion strains. Micrographs show the response of *rsxD* and *rsxG* deletion strains and the double deletion strains Δ*rsxD soxS:Km* and Δ*rsxG soxS:Km* on cephalexin (200 μg/ml) containing agar pads. A *soxS* deletion in *rsxD* and *rsxG* mutants leads to faster lysis. Scale bar corresponds to 20 μm.

To understand which cellular functions could play a role in modulating lysis kinetics, we performed a network analysis (see section “Materials and Methods” for details) of the deleted genes in the tolerant strains using an adapted version of Phenetic (De Maeyer et al., 2015). We picked 668 strains (Supplementary Data Sheet S2) that showed more than 10-fold enrichment (or SI > 0.43) in the fraction of intact cells at T_2__h_ compared to the wild type for the analysis. Phenetic integrates multiple levels of molecular interaction data such as protein-protein, protein-DNA, and metabolic interactions to search for either regulatory mechanisms or pathways that explain the phenotype. Several clusters of proteins emerged from the analysis of the tolerant strains (Supplementary Figure S7). One cluster is composed of the twin-arginine translocation (Tat) protein export system (TatA, TatB, and TatC) and two of its substrates – FtsP and CueO. Tat pathway also exports amidases AmiA and AmiC to periplasm in *E. coli* (Bernhardt and de Boer, 2003). The filamentation phenotype of *tat* deletion strains could be due to defects in export of amidases. Deletion of genes coding for the outer membrane porins OmpA, OmpF, and PhoE caused tolerance to β-lactams. Surprisingly, three clusters were composed of motility-associated proteins. Deletion of flagellar genes (*fliJ*, *fliI*, *flgA, flgE, flgC, flgK*, and *fliD*), motility-associated genes (*cheW*, *cheA*, and *cheZ*), and type IV pili genes (*hofQ*, *hofC*, *hofB*, and *ycgR*) delayed lysis. Deletions in the *rsxABCDGE* gene operon also inhibited lysis (Supplementary Figure S7). Deletion of genes coding for Rsx proteins initiates the SoxS response in *E. coli* (Koo et al., 2003). SoxS activation leads to lower accumulation of drugs inside the cells due to increased efflux by the AcrAB efflux pump and decreased influx through down-regulation of OmpF (Miller and Sulavik, 1996). Since decreasing the intracellular concentration of cephalexin can essentially delay the onset of lysis (Supplementary Figure S1), we hypothesized that the SoxS response could explain the late lysis of *rsx* deletion strains. We constructed Δ*soxS*Δ*rsxD* and Δ*soxS*Δ*rsxG* to verify that *soxS* deletion affects the lysis kinetics of the *rsx* strains. Both double deletion strains lysed faster than their corresponding single deletion strains (Figure 7).

In summary, the network analysis pinpointed various protein complexes and cellular processes that can alter the cephalexin induced lysis kinetics. Further study of these will broaden our understanding of antibiotic tolerance mechanisms. Since lysis kinetics is dependent on the concentration of cephalexin, it is not surprising that many gene deletions such as *ompA*, *ompF*, and *phoE* that may impair the uptake of cephalexin also slow down lysis. Deletion of Rsx proteins caused tolerance in a SoxS-dependent fashion. This suggests that the activation of SoxS response in *rsx* deletion strains could be lowering the concentration of cephalexin. Mutants with known daughter cell segregation defects like Δ*dedD*, Δ*envC*, and Δ*tatA/B/C* (Rodolakis et al., 1973; Stanley et al., 2001; Gerding et al., 2009) were also found to confer late lysis (Supplementary Figure S8), highlighting the importance of cell wall remodeling during division to the process of lysis.

## Conclusion

β-lactam antibiotics exhibit time-dependent killing and the time during which the concentration of the antibiotic stays above the MIC is a major determinant of their antibacterial activity (Vogelman et al., 1988; Turnidge, 1998). Antibiotic tolerance increases the time needed for sufficient antibacterial action and if this tolerant phenotype goes unnoticed, β-lactam treatment may fail due to insufficient time above MIC. A systematic genome-wide phenotypic analysis of β-lactam tolerance was hitherto difficult due to lack of a suitable high-throughput assay for measuring killing kinetics. We applied a recently developed methodology for high-throughput microscopy of bacteria grown in 96-well plates (Zahir et al., 2019) and measured lysis kinetics of all mutants in the Keio collection (Baba et al., 2006). The image-based dynamic phenotyping of the response to β-lactams revealed several proteins that modulate the process of lysis. The late lysing strains show filamentation-mediated tolerance. Mainstream antibiotic susceptibility assays like MIC or growth fitness on agar pads containing sub-inhibitory antibiotics cannot identify filamentation-mediated tolerance. We also identified *tolQ*, *tolR*, and *pal* deletion in *E. coli* leads to rapid lysis in response to PBP3 inhibitors. We hope that this study serves as a trigger for similar high-throughput investigations of antibiotic tolerance mechanisms, ultimately leading to the development of novel strategies like antibiotic dosage regimes and drug combinations for guiding antibiotic therapies.

## Materials and Methods

### Bacterial Strains and Growth Conditions

High-throughput microscopy screening was performed using the ordered Keio collection of non-essential single-gene deletion mutants. In the entire manuscript, *E. coli* BW25113 is referred to as the wild type. All experiments were conducted at 37°C in LB medium. All the strains studied in detail were also derived from the same library. The kanamycin resistance cassette from candidate strains was reintroduced into the wild type by P1*vir* phage transduction (Thomason et al., 2007). The resistance cassette was excised using the plasmid pCP20 and following the protocol described in Datsenko and Wanner (2000).

For complementation assays, the protein coding regions of MrcB, YcfM and YbgC was inserted in the multiple cloning site of pBAD vector. The genes were amplified from *E. coli* BW25113 genomic template DNA using the primers given in Table 1 to generate fragments with *Sac*I-*Pst*I sticky ends. The fragment was cloned into the *Sac*I-*Pst*I double digested pBAD vector using Gibson assembly protocol (Gibson et al., 2009).

**TABLE 1:** Primers for amplifying the candidate genes.

**Primer name**	**Sequence**
mrcB1	tcgatggggatccgagctaggaggaattcaccatcatggccgggaatgaccg
mrcB2	catatggtaccagctgcattaattactaccaaacatatccttgatcca
lpoB1	tcgatggggatccgagctaggaggaattcaccatcatgacaaaaatgagtcgctacgc
lpoB2	catatggtaccagctgcattattgctgcgaaacggcac
ybgC1	tcgatggggatccgagctaggaggaattcaccatcgtgaatacaacgctgtttcga
ybgC2	catatggtaccagctgcatcactgcttaaactccgcga

### High-Throughput Microscopy Screening and Analysis

The imaging methodology used in this study was recently developed in our lab, details of which will be published elsewhere (Zahir et al., manuscript under review). Briefly, strains were grown overnight in 96-well plates (Greiner, 96 Well Polystyrene Microplate, clear) containing LB with 30 μg/ml Kanamycin (MP Biomedicals), and then diluted 5000 times in a 96-well plate with glass bottom (Brooks Automation Ltd., Ref. MGB096-1-2-LG-L) containing fresh media. After 2 h of additional growth all strains were treated with 200 μg/ml (final concentration) of cephalexin (Sigma-Aldrich). After adding the antibiotic, the plate was kept on the microscope at 37°C for imaging. The screen was performed with a Nikon Ti-E inverted microscope equipped with Qi2 CMOS camera and temperature-controlled cage incubator. Images were acquired using the CFI Plan Apochromat Lambda DM 40X air objective (NA = 0.95). A custom pipeline of macros implemented in Nikon microscopy software was used to control automated movements of stage and objective for image acquisition. A 5-min idle time was placed before acquiring the Z-offset settings to let the cells settle down at the bottom. For each time-point, we took 9 images from every well. The time gap between first image (well A1) and last image (well H12) is 16 min. The images were analyzed using the Nikon NiS-Elements software. The intact cells were segmented using thresholding and then counted. On average 226 cells were imaged for each mutant at T_40__*min*_. Wells with less than 20 cells were discarded from the data analysis and the experiment was repeated for those strains. Minimum cell size was set to 1 μm for first time-point, 3 μm for second time-point and 5 μm for third and fourth time-point. Survival Indexes (SI) for T_85*min*_ were calculated by dividing the number of cells at T_85__*min*_ by the number of cells at T_40__*min*_. SI for T_2__h_ and T_4_._5h_ were calculated by dividing the number of cells at the respective time-points by the number of cells at either T_40__*min*_ or T_85__*min*_ (whichever is more). We kept one well containing the wild type in all experiments for quality control (Supplementary Figure S8). For every batch, we verified that the wild type started lysing before T_85__*min*_ and most of the wild-type cells lysed by T_2__h_.

### Time-Lapse and Fluorescence Imaging

Time-lapses of wild type and mutants that showed late or early killing kinetics with cephalexin were done on LB-agarose (2%) pads containing the appropriate amount of cephalexin. Strains were grown overnight, then diluted 1:1000 next day and grown until they reached an OD_595_ of around 0.25. Then cells were seeded on the agarose pads for imaging. For labeling the cell wall we used 25 μg/ml of Wheat Germ Agglutinin, Tetramethylrhodamine conjugate (Thermo Fisher Scientific). For showing the physiology of filamented cells, an overnight culture of the Δ*fkpB* was diluted and grown to 10^6^ CFU/ml and then treated with cephalexin (200 μg/ml) for 2 h. Subsequently, a 1 ml aliquot was washed with PBS buffer to remove the antibiotic and then stained with DAPI (4’,6-diamidino-2-phenylindole) from Sigma-Aldrich. DAPI staining was performed as described before (Dewachter et al., 2017). To show recovery of filamented cells on agar pads cell cultures were treated with appropriate amount of cephalexin for 2 h and then the antibiotic was washed away. 25 mM MgSO_4_ was added to the media during centrifugation and washing to prevent lysis. Cells were then seeded on LB-agarose pads for microscopy. Cell wall morphology experiments were done on a Zeiss Axio Imager.Z1 fluorescence microscope equipped with an EC Plan-NEOFLUAR 100X objective. All the other images were taken on a Nikon Ti-E inverted microscope.

### Network Analysis of Tolerant Strains

Network-based analysis was performed using an adapted version of PheNetic (De Maeyer et al., 2015). First, the deleted genes that led to a significant increase of survival rate (SI > 0.43, which represents a 10-fold enrichment in survival rate when compared to the wild type) were mapped on the interaction network. The interactions of the interaction network were then weighted based on (1) the network topology to reduce the effect of large hubs and (2) the mean survival rate of the deleted genes which are on either end of the interaction. If either gene was not in the mapped deleted genes list, its survival rate was set to zero, meaning it can still be picked by the analysis as it can be part of an interesting molecular pathway even if knocking out the gene reduces the survival rate of the organisms or if it was not possible to assess the deleted gene. A subnetwork of the interaction network which contains the molecular pathways which are most probable to be involved in cephalexin tolerance is finally inferred by performing a pathfinding and subsequent inference step using the weighted network. For the technical details on the pathfinding and inference steps we refer to De Maeyer et al. (2016). The parameters given to PheNetic in order to obtain the results were the following: -y 0.025 -k 0.5 -c 0.025 -r 5 -b 35 -× 150. This means that first the cost parameter was swept from 0.025 to 0.5 with steps of 0.025, each optimal subnetwork was calculated 5 times before taking the best result (from these 5) as the resulting subnetwork for that specific cost, secondly the maximum size of a resulting subnetwork was 150. Larger resulting subnetworks were discarded as they are hard to interpret and contain less interesting results. Finally, the number of N-best paths was set to 35.

The interaction network for *E. coli* K-12 W3110 used in the network-based analysis was obtained from STRINGdb version 10.5, KEGG release 84.1 and RegulonDB release 9.4. Metabolic, (de)phosphorylation and (de)methylation interactions were obtained from KEGG, protein-protein interactions were obtained from STRINGdb in which only interactions with a combined score of at least 800 were retained and regulatory interactions were obtained from RegulonDB in which TF-gene, TF-TF, and sigma factor interactions were considered. This resulted in an interaction network consisting of 3941 genes and 25672 interactions between these genes. When mapping the genes list to this network, 500 out of 668 deleted genes could be mapped (around 89%).

### Turbidimetric Studies

Optical density measurements at 595 nm before and after the antibiotic treatment were taken using the Synergy Mx Multi-mode Microplate Reader (Biotek). Overnight grown culture of the wild type was diluted 1:100 and grown in 96-well plates. After 2 h of growth, different quantities of cephalexin were added to the wells.

### Killing Kinetics and Measurement of Viability

For time-kill assays, overnight samples were diluted 1:5000 in fresh media and when the cell density reached around 10^6^ cells/ml, the cultures were treated with cephalexin 50 or 200 μg/ml. Aliquots of 1 ml were taken at appropriate time-points and washed in 25 mM MgSO_4_ solution. Cells were then serially diluted and plated on LB agar plates supplemented with 25 mM MgSO_4_. Cell survival was measured by CFU counts. All the experiments were repeated thrice.

### MIC and MBC Determination

Minimum inhibitory concentrations and minimal bactericidal concentrations were both determined at the same time by a broth micro-dilution method (Wiegand et al., 2008). Briefly, an overnight culture was diluted in LB to an inoculum of approximately 5 × 10^5^ CFU per ml, incubated in a range of two-fold antibiotic dilutions and grown for 18–24 h. After incubation, the absorbance at 595 nm was measured. Afterward, the cultures of the wells that showed no growth were plated out. The MBC was defined as the lowest antibiotic concentration that killed 99.9% of the initial number of the cells in the inoculum. This was determined by plating out the cultures of the wells that showed no growth during MIC determination.

### Doubling Time Measurements

All strains were grown overnight in 3 replicates. Next day they were diluted 5000 times in fresh LB and grown for 2 h to approximately 10^6^ CFU/ml. Aliquots of 1 ml were taken and serially diluted and plated on LB agar plates. Cultures were grown for 1 h more and then again plated out. Doubling time was computed from the CFU counts obtained in the two time-points.

### Spot Titer Assay

Strains were grown overnight in test tubes with 5 ml LB. The next day, cultures were normalized to OD_600_ = 0.1 and then serially diluted. The serial dilutions were spotted (5 μL) on the agar media and the plates were incubated at 37°C overnight (16–20 h).

## Data Availability Statement

All datasets generated for this study are included in the article/Supplementary Material.

## Author Contributions

TZ, MF, and JM conceived the work, designed the experiments and interpreted the data, and wrote the manuscript. TZ designed the high-throughput imaging setup, performed the screening, analyzed the data, and performed additional experiments. SF helped with the antibiotic susceptibility assays. DW performed the complementation experiments. RC helped with the imaging setup. BW performed the gene network analysis. All authors reviewed and contributed to the final version of the manuscript.

## Conflict of Interest

The authors declare that the research was conducted in the absence of any commercial or financial relationships that could be construed as a potential conflict of interest.
