# Supplementary material for: Image-Based Dynamic Phenotyping Reveals Genetic Determinants of Filamentation-Mediated β-Lactam Tolerance
Source: Front Microbiol. 2020 Mar 13;11:374. doi: 10.3389/fmicb.2020.00374 (PMC7082316; doi:10.3389/fmicb.2020.00374)
Supplement: Supplementary file 1 [file Data_Sheet_1.docx]

Supplementary Material

# Supplementary Figures and Tables

## Supplementary Figures


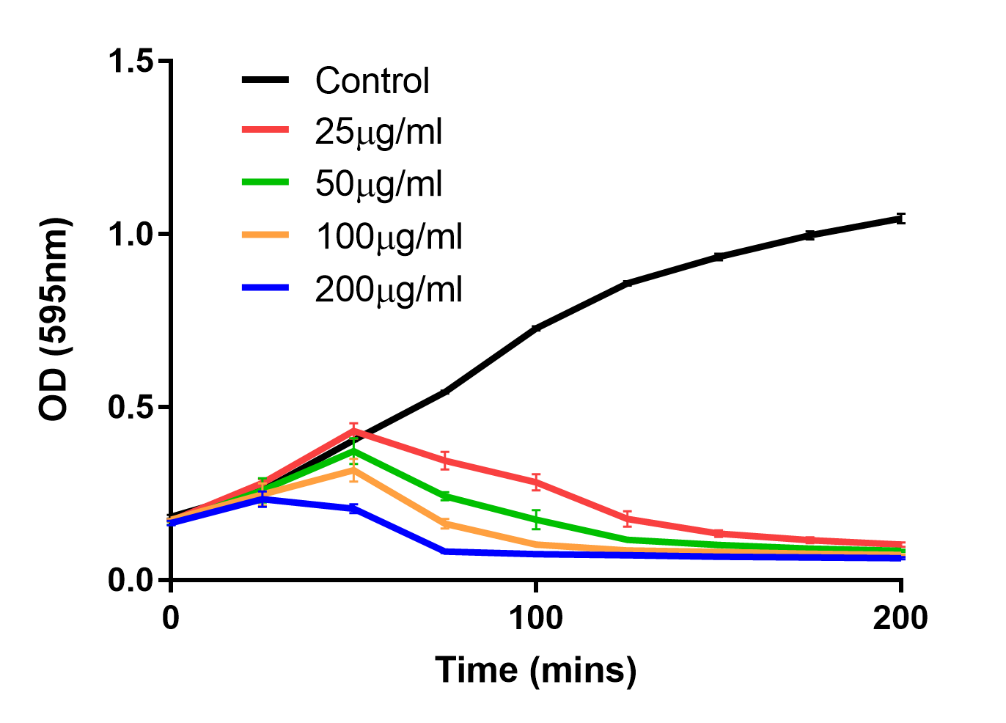


**Supplementary Figure 1: Lysis kinetics of the wild type at different concentrations of cephalexin.** Wild-type *E.coli* was grown in 96-well plates (Greiner, polystyrene clear flat bottom plates) and treated with different concentrations of cephalexin at time = 0. OD was measured to follow the progress of lysis. Increasing the concentration of cephalexin leads to faster lysis. All data points correspond to 3 replicates.


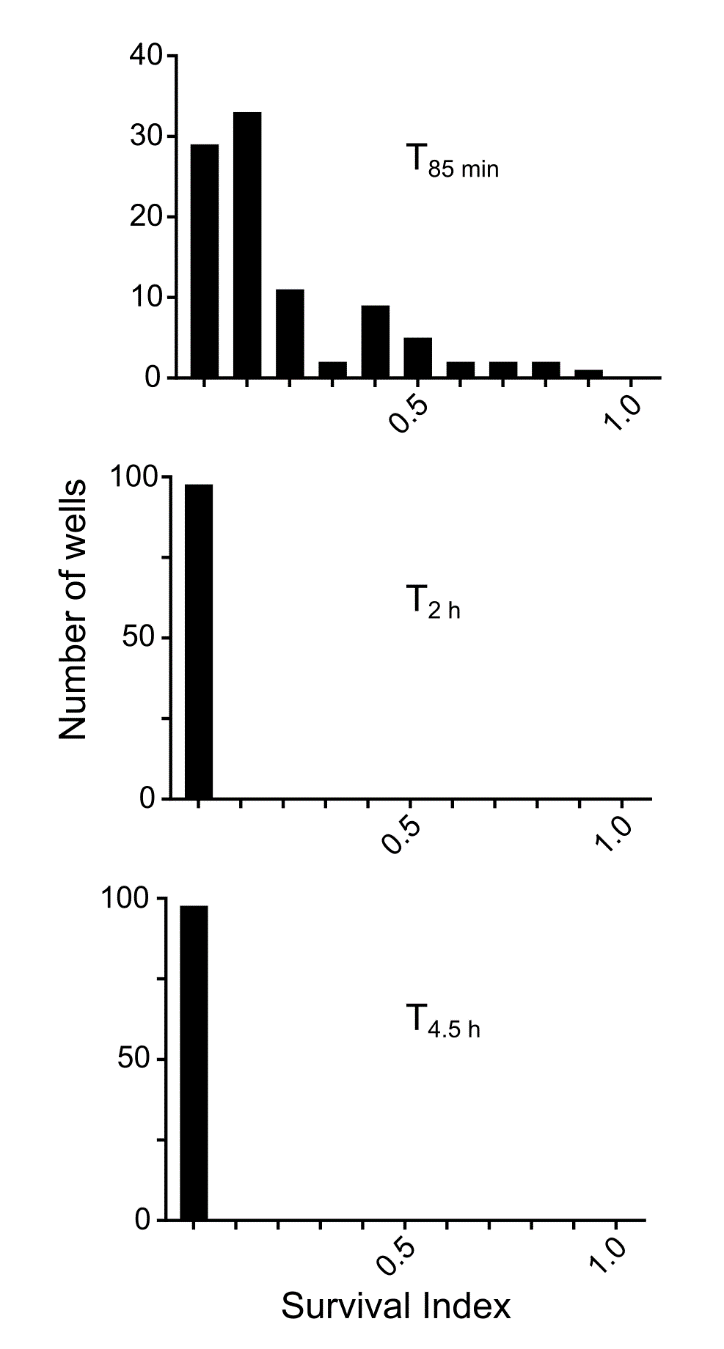


**Supplementary Figure 2: Lysis kinetics of the wild-type *E. coli*.** Distribution of survival index (SI) for 96 replicate cultures of the wild type treated with cephalexin 200μg/ml. The SI is calculated by taking the ratio of intact cells at each time-point to the number of intact cells segmented at a previous time-point (T_40 min_ or T_85 min_; whichever is more).


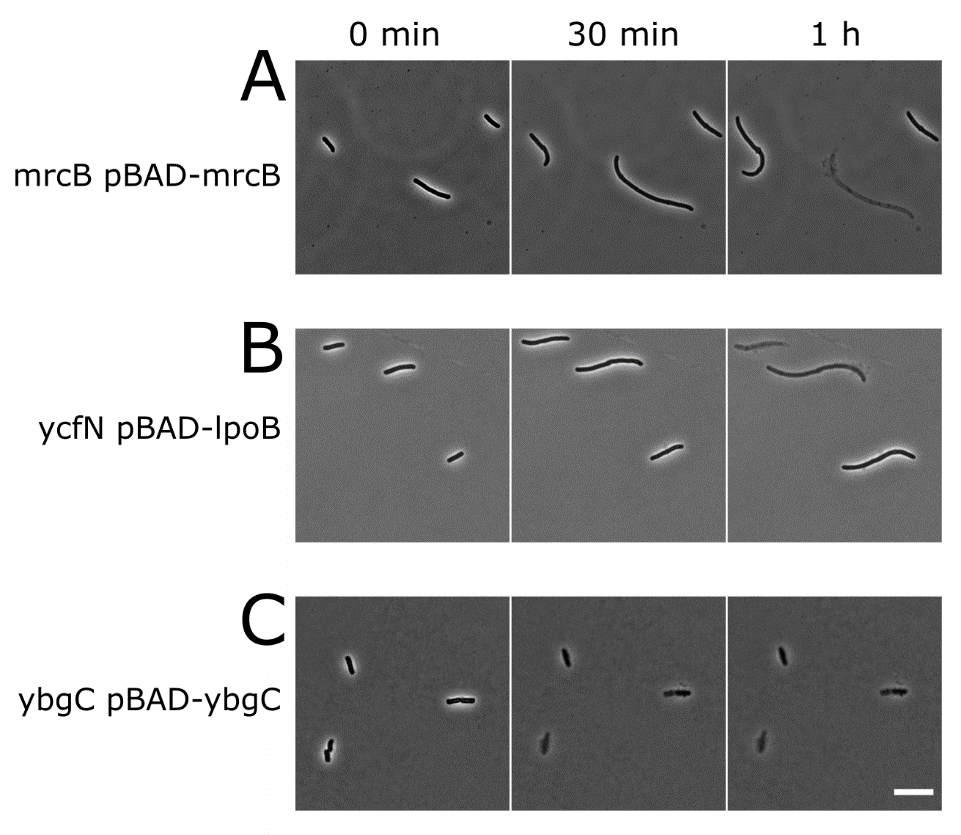


**Supplementary Figure 3: Complementation of rapidly lysing strains.** Shown are micrographs of strains grown to early exponential phase and seeded on agar containing 50µg/ml cephalexin. (A) Trans expression of *mrcB* reverts the lysis phenotype of Δ*mrcB* to the wild type. (B) Trans expression of *lpoB* reverts the lysis phenotype of Δ*ycfN* to the wild type. (C) Trans expression of *ybgC* does not affect the rapid lysis phenotype of Δ*ybgC*. Arabinose 0.2% was used to induce the expression. The scale bar corresponds to 10 μm.


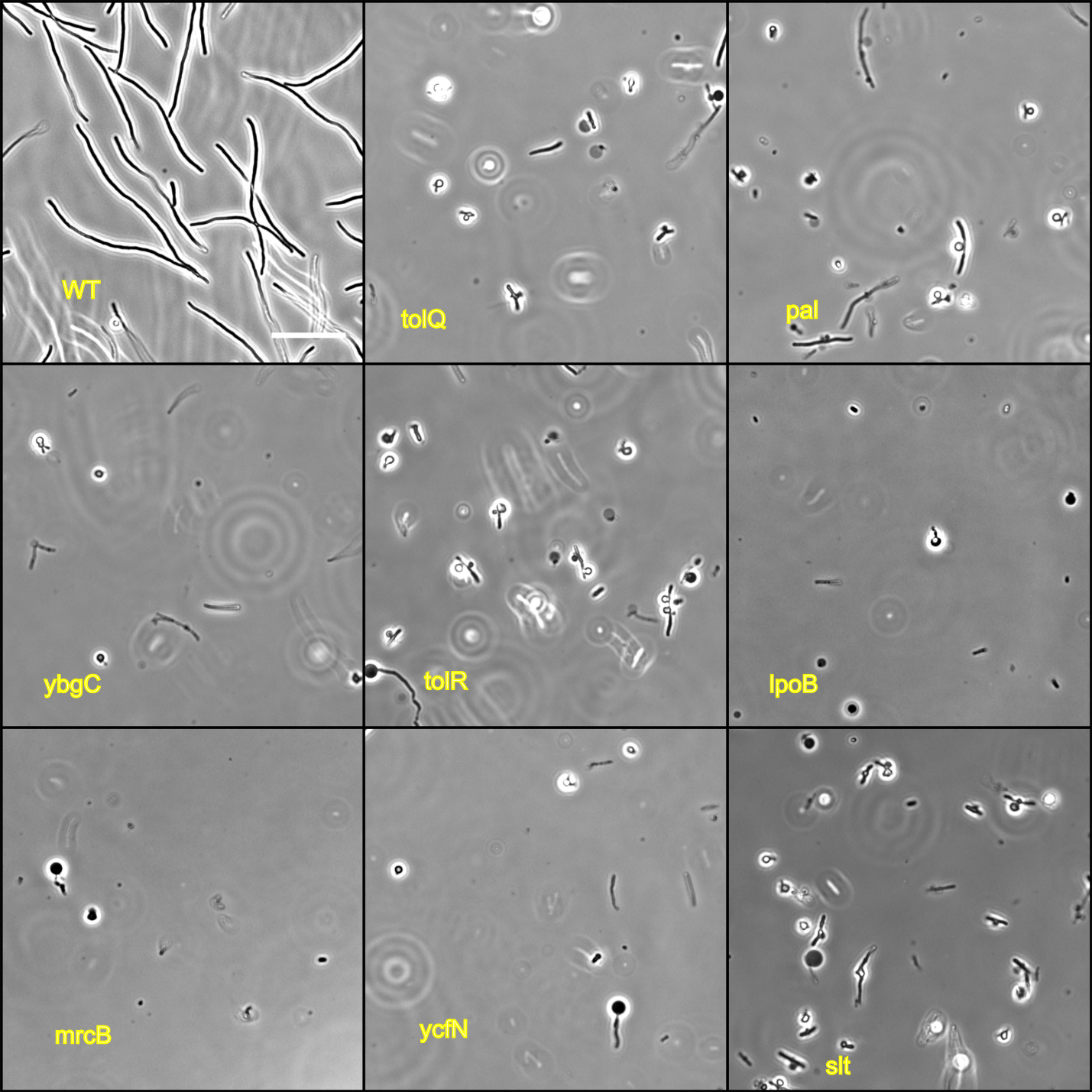


**Supplementary Figure 4: Response of rapidly lysing *E. coli* strains to ceftazidime.** Micrographs of the wild type and all of the rapidly lysing strains after 1 hour of exposure to ceftazidime 2μg/ml are shown. The wild type shows smooth and long cells while the rapidly lysing strains show lysed and bulging cells. The scale bar corresponds to 25 μm.


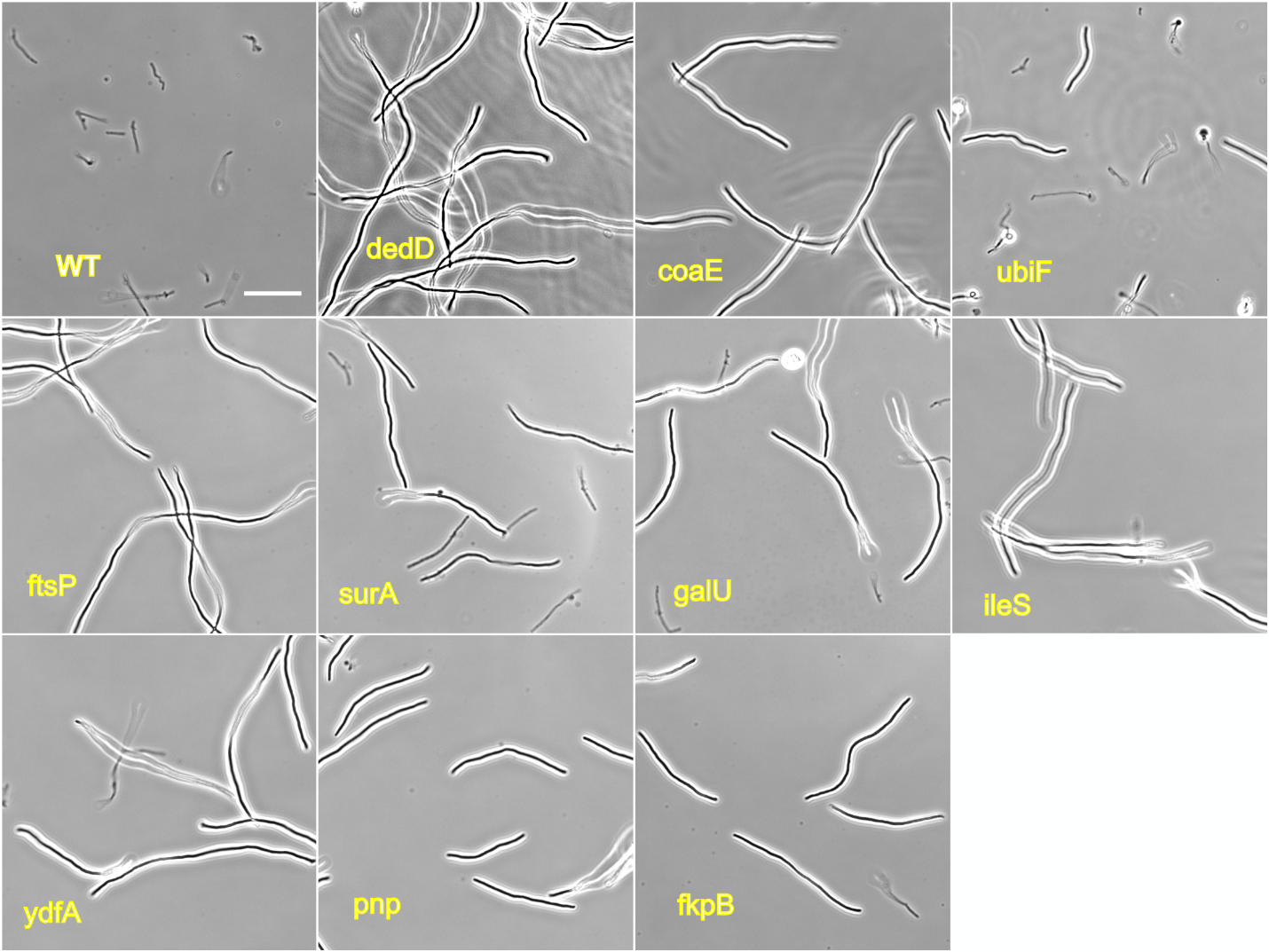


**Supplementary Figure 5: Response of late lysing strains to ceftazidime.** Micrographs of the wild type and all of the late lysing strains after 2 hours of exposure to ceftazidime 20 μg/ml. Scale bar corresponds to 25 μm.


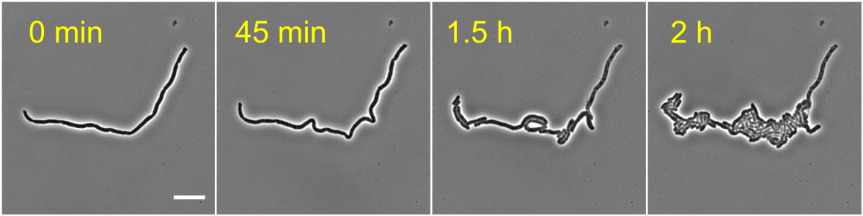


**Supplementary Figure 6: Filamented wild-type cells can recover from antibiotic exposure to form a colony of normal cells.** Shown is a time-lapse of a wild-type cell recovering after 2 hours of exposure to cephalexin (16 μg/ml). Scale bar corresponds to 10 μm.


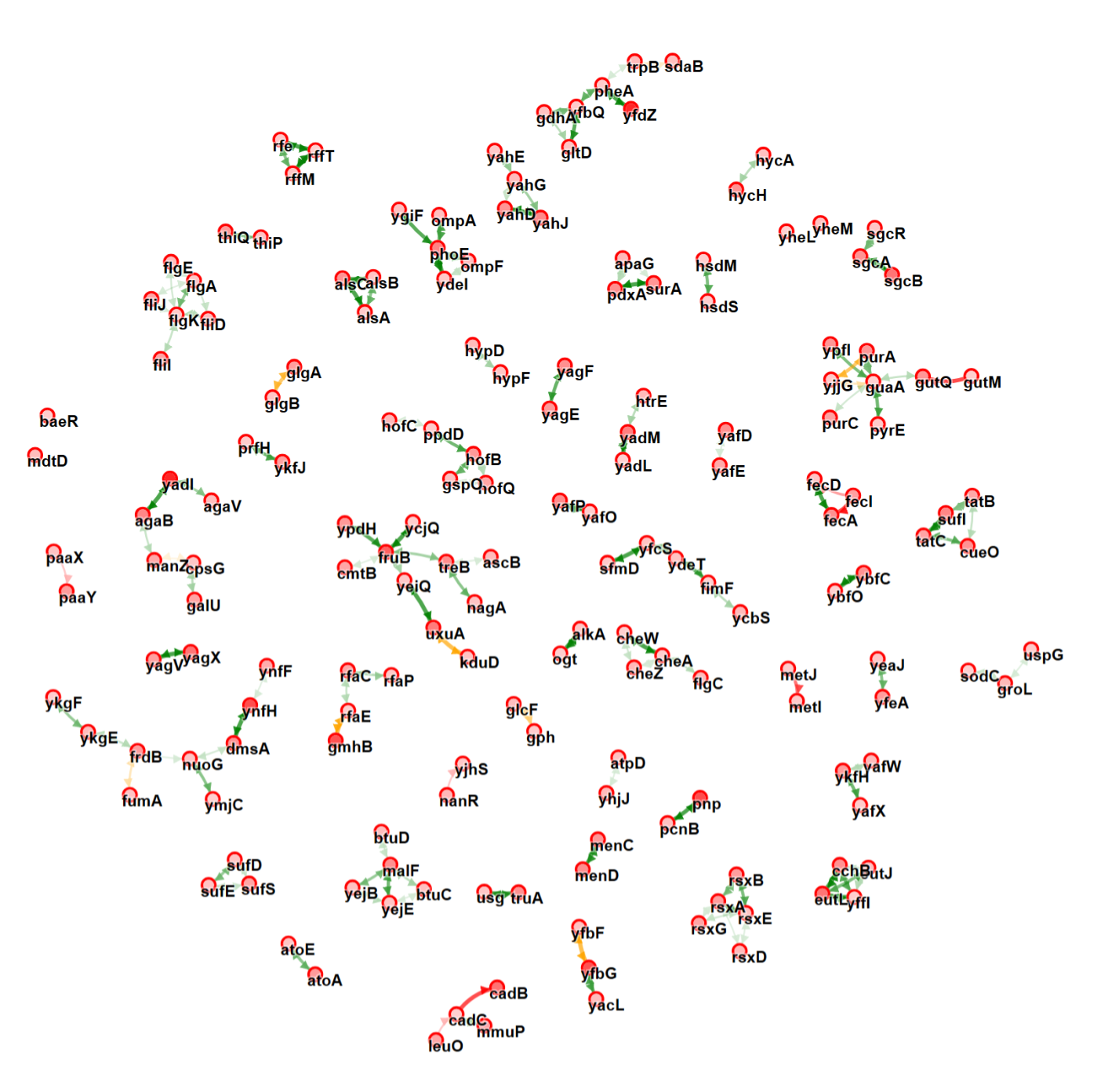


**Supplementary Figure 7: Network analysis of 674 proteins corresponding to gene deletions in *E. coli* mutants that showed more than a 10-fold enrichment in the fraction of intact cells 2 h after treatment with cephalexin. The r**esulting subnetworks were obtained using PheNetic. Green edges represent protein-protein interactions, red edged show repressing interactions, yellow edges represent metabolic interactions. The opacity of the edges represents the degree of belief that these interactions play a role in the underlying phenotype where more opaque edges represent a higher degree of belief. The nodes represent genes/gene products. The inner color of a node represents the SI values. Dark red means higher value of SI. Grey nodes were not assessed in the experimental setup.


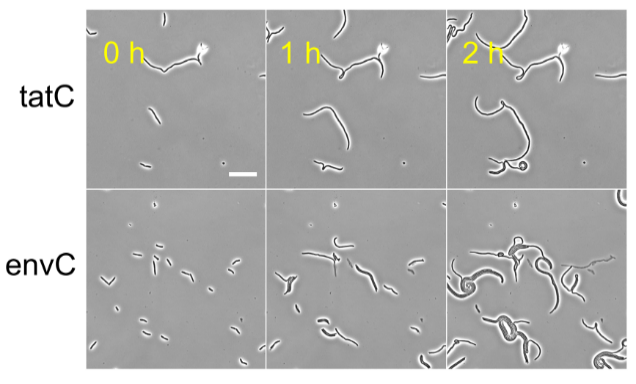


**Supplementary Figure 8: *tatC* and *envC* deletion delays lysis in the presence of cephalexin.** Time-lapse microscopy showing the response of *tatC* and *envC* deletion strains on 200 μg/ml cephalexin containing agar pads. Scale bar corresponds to 25 μm.

## Supplementary Tables

**Supplementary Table 1.** Doubling times of *E. coli* strains (min). The average and standard deviation is computed from 3 replicates.

| Strains | Average doubling time (min) |
| --- | --- |
| Wild type | 19.65 ± 1.42 |
| Δ*pnp* | 28.27 ± 3.78 |
| Δ*fkpB* | 20.85 ± 3.38 |
| Δ*ubiF* | 35.70 ± 5.66 |
| Δ*surA* | 19.02 ± 1.21 |
| Δ*dedD* | 20.6 ± 1.3 |
| Δ*galU* | 21.56 ± 2.3 |
| Δ*ftsP* | 19.14 ± 1.71 |

**Supplementary Table 2.** Cephalexin MIC and MBC values in μg/ml

| Strains | MIC (μg/ml) | MBC (μg/ml) |
| --- | --- | --- |
| Wild type | 8 | 16 |
| Δ*pnp* | 8 | 8 |
| Δ*fkpB* | 8 | 16 |
| Δ*ubiF* | 16 | 16 |
| Δ*surA* | 8 | 16 |
| Δ*dedD* | 8 | 16 |
| Δ*galU* | 16 | 16 |
| Δ*ftsP* | 8 | 16 |
